# Defining sports moral character and clarifying its related concepts

**DOI:** 10.3389/fspor.2023.1101377

**Published:** 2023-02-17

**Authors:** Cong Liu, Guofeng Qu, Rong Gao

**Affiliations:** College of P. E and Sports, Beijing Normal University, Beijing, China

**Keywords:** sports moral character, sports morality, sports character, sportsmanship, concept

## Abstract

This study examines the concept of sports moral character and clarifies the differences between it and related moral concepts in sports. The research is conceptual and uses the methods of a literature review and logical analysis. Sports moral character is shown to have the characteristics of practicality, growth, and integration. It is a stable moral quality that is gradually formed and displayed in sports practice under the influence of family, school, and social environments. Sports moral character differs in some ways from other related concepts. Sports morality is the objective existence of “reason,” to which sports character and sportsmanship are both more applicable than is sports moral character.

## 1. Introduction

A concept is the logical starting point for both thinking and research. In previous studies, common colloquial terms such as sportsmanship (or sportspersonship), character, or fair play have been used to describe moral development in sports (1). These words can be used to describe participants’ good or bad moral psychologies and behaviors in sports. However, as vocabulary usage scenarios have become increasingly complex, vocabulary usage habits have gradually changed. Shields and Bredemeier (2) noted that the concepts of morality and moral education have been employed with decreasing frequency, whereas the concepts of character and character education have been increasingly utilized. Moreover, many studies have begun to focus on the results of moral character development (3–6). This phenomenon is also occurring in the field of sports, but new and old words have not been defined or differentiated, which has caused confusion in the theoretical and practical research of moral development in sports. Especially as regards the sports participation of young students and athletes, poor guidance may lead to an improper development. For example, some PE teachers may cultivate students’ sports moral character by creating a series of game rules that reward “champions”; Many coaches often regard social character content, such as persistence and struggle, as the core of moral character development (7).

How can we define sport moral character? What are the differences between sports moral character and sports character, sports morality, and sportsmanship? Concept research is a new approach that can further the development of sports moral character. This study defines and differentiates the characteristics and connotation of sports moral character and related concepts to provide a reference for the theoretical research on and the teaching practice of sports moral character.

## 2. Methods

In this study, a literature research focuses on a conceptual analysis. To define the relationship between sports moral character and related concepts, the keywords “sports morality,” “sportsmanship,” “sports moral character,” and “sports character” were used in electronic databases such as Web of Science, ProQuest, Scopus, EBSCO, and Google Scholar. The retrieved literature on sports moral character and related concepts was read, and the concepts were classified and summarized.

## 3. Interpretation of moral character

To understand the connotation of sports moral character, we should determine the origin of “moral character” and understand its characteristics. The notion of moral character can be traced to Aristotle's Nicomachean Ethics (8), in which Aristotle used the word “hexis” to express a habit that involves knowledge and moral character. Aristotle indicated that a good person must have a moral character and knowledge to choose and practice good habits (5). Using Aristotle's threefold division of the things that exist in the soul (potency, habit, and emotion), George (9) defined a moral character as the sum of a person's moral habits and dispositions. This definition is consistent with the understanding of many other researchers. Pervin (10) defined a moral character as a disposition to express behavior in consistent patterns of functions across a range of situations. Cohen and Morse (11) adopted the three-factor model (motivation, ability, and identity) and maintained that a moral character can be conceptualized as an individual's disposition to think, feel, and behave in an ethical vs. an unethical manner.

Thus, a moral character has at least the following three characteristics. First, its subject is the individual. Second, it should be related to moral values. Third, it is a stable disposition through which behavior is expressed. Because of its first characteristic, the concepts of a moral character can be clearly distinguished from morality. Morality is a kind of norm referring to a code of conduct that, given specified conditions, would be upheld by all rational people (12). A moral character is found in an individual's soul. It is a personalized form of morality, and it consists of the transformation of morality from social external norms to individual, internal psychology. In addition, while a moral character has both psychological and moral characteristics, it differs from the general psychological quality of individuals. An ancient states, “Those who have virtue also obtain it. … They obtain it from themselves, and they obtain it from others.” In Asian culture, the core concept of the term “moral character” is “Te (德),” whose connotation (e.g., 德, moral) is connected with “De (得, gain)”, which means that moral characters and moral behaviors should benefit both ourselves and others (13). In this context, the personal quality characteristics of “oneself” may not necessarily be moral. For example, a person who is overly self-centered is regarded as selfish, while a person who is brazen in doing evil things may be antisocial.

In addition, the characteristics of personal psychological quality are closely related to character. Here, it is necessary to clarify the terms “moral character” and “character.” A Dictionary of Philosophy defines “character” as the sum of a person's dispositions to action (14). Moreover, the term character is often used in character education. Character education began in the United States at the end of the 20th century. It has a long developmental history and is the mainstream method of moral education in the United States. A character education partnership defines character education as the conscious efforts of schools, families, and communities to help children understand, cultivate, and practice core ethical values (15). From this perspective, character includes both moral character and the ability to achieve moral values. In Berkowitz and Grych's (16) definition of character, character has four foundational components (social orientation, self-control, compliance, and self-esteem) and four moral components (empathy, conscience, moral reasoning, and altruism). These classification methods are widely recognized and applied in character education. Lickona and Davidson (17) proposed that character includes performance character and moral character. Rudd and Stoll (7) noted that character can be divided into social character and moral character. Shields (18) advocated that K-12 education should help children develop four kinds of character: intellectual, moral, civic, and performance. Therefore, many experts agree that moral character is included in character.

However, although social character (or performance/intellectual/civic character) is necessary for social life, it can be used as a support for moral character. Can moral character affect the development of social character? How does moral character affect the formation of character? Few studies have fully explained these problems. Shields (18) claimed that moral character cannot be reduced to a “bag of virtues,” and the goal of character education is to develop a disposition to seek goodness. Baehr (19) noted that moral character can be considered a concern with distinctively moral goods such as the alleviation of another person's suffering. Such concerns can be seen as another important feature of moral character, which can be called moral coordination. In the Tao Te Ching, Laozi wrote “Tao” begets them. “Te” nurtures them. … Therefore all things respect not only “Tao” but also “Te” (20). The “Tao” mentioned here can be understood as the universe, while “Te” is morality, and the internal perspective of the individual is moral character. Lao Tzu believed that the highest virtue is to let things exist and develop in the most reasonable way. Moreover the I Ching (21) states that if “Te” does not match a person's position, disaster will result. This statement shows that, for a person to have a good moral character, he or she must behave well and achieve the moral purpose that is within his or her ability. In general, these thoughts all explain that individual moral character plays the role of coordinating morality in the development of things.

In summary, a moral character can be defined as a stable disposition of an individual in the process of coordinating the practice of a moral purpose. Under the moral practice in different social contexts, a moral character plays a role in coordinating the accomplishment of moral purposes. In this process, the practice of moral behavior needs the support of social character, and the practice can affect the development of individual social character and ability, thereby forming one's character.

## 4. Concept and characteristics of sports moral character

### 4.1. Definition of sports moral character

Definition is a logical way to clarify a concept, and the most common definition method is genus plus species difference, for which the general formula is that the “defined item is equal to species difference plus the adjacent genus concept” (22). That is, the genus concept and species difference with the smallest extension adjacent to the defined item constitute the definition. Here, sports moral character is the defined item. In the above analysis of moral character, character is the word with the closest meaning to moral character. By extension, in the sports context, sports character can be regarded as the closest concept to sports moral character. In addition, sports social character and sports moral character are components of sports character, but each differs from the other. In short, the essential difference between sports social character and sports moral character is that the former are “not directly related to morality”. This topic is discussed in detail in what follows.

### 4.2. Characteristics of sports moral character

Sports moral character is the embodiment of moral character in the sports field; in fact, it is a subconcept of moral character. Understanding the characteristics of sports moral character is helpful for evaluating and cultivating it. Sports’ forms and characteristics demonstrate that sports moral character has the characteristics of practicality, growth, and integration.

#### 4.2.1. Practicality of sports moral character

Sports can positively affect different cognitive processes (23) and executive functions (24, 25) and promote the development of personal and social responsibility (25–27). However, these practices are based on participation in physical activities. Therefore, it is impossible to talk about the practice of sports moral character without addressing the body's participation. Here, the practicality of sports moral character can be highlighted because only when people actively train and compete can they gradually form a stable sports moral character. A weak and passive person may have difficulty demonstrating a good moral character in sports contexts. In addition, different sports have different physical and mental practice requirements. Even under the rules of the same sport, different roles have different requirements. Brenda et al. (28) noted that each sport has its own implicit culture and ethics. The rule structures of various sports activities promote different types of social communications. All sports can provide an opportunity for participants to develop their moral character, although these impacts vary among different kinds of sports. Yaffe et al. (29) noted that participants in team sports tend to tolerate more cheating and gamesmanship than do participants in individual sports. Team (vs. individual) athletes tend to value status in sports relatively more, while individual (vs. team) athletes tend to value competence relatively more. In addition, Yaffe et al. (29) also highlighted the differences among individual sports. Sanlav and Donuk (30) found that track and field as well as tennis athletes demonstrate more integrity, compassion, sportspersonship, and fairness than do other athletes. Suwiwa et al. (31) reported that sport science activities such pencak silat, swimming, dance, and outdoor activities can improve character in various ways. From this point of view, school physical education is not limited to a certain sport, so such education can provide more opportunities for the practice of sports moral character. Burgueño and Medina-Casaubón (32) assumed that one of the main goals of physical education is to develop the students’ moral and ethical domains. Laker (33) noted that physical education should contribute to the development of the whole person—including physical, emotional, social, and personal (or affective) outcomes—as well as spiritual and community development.

#### 4.2.2. Growth of sports moral character

The attitude and ability of individuals to participate in sports often change over time, as does their sports moral character. Piaget's moral cognitive development theory holds that the stages of individual moral development include lawless, heteronomy, self-discipline, and justice (34). Based on this theory, Kohlberg (35) proposed the theory of three levels and six stages of moral development. He believed that moral development could be divided into the preconventional, conventional, and postconventional levels; furthermore, each level could be divided into two stages. The preconventional level includes the punishment and obedience orientation stage and the relative utilitarian orientation stage. The conventional level includes the seeking recognition orientation stage and the abiding by laws and regulations and order orientation stage. The postconventional level is divided into the social contract orientation stage and the principle or conscience orientation stage. In addition, these theories continue to have age characteristics in the division of different stages, which is closely related to individuals’ physical and mental development.

The development of sports moral character is a similarly gradual growth process. Throughout primary school, middle school, university, and beyond, as individuals accumulate knowledge and skills and enrich and shape their values from sports, students’ moral character is constantly endogenous, gradually formed, and stable. Moreover, individuals are affected not only by their own physical and mental development but also by the moral practice experienced in society and in the stadium. As the sports moral cognition and the moral practice ability of sports participants develop, so does their sports moral character; that is, sports moral character demonstrates a growth characteristic.

#### 4.2.3. Integration of sports moral character

Obviously, individuals must have a universal social moral foundation before participating in sports; an individual's original social moral consciousness will directly or indirectly interact with the sport's code of conduct. After participating in the sport for some time, one's individual experience forms a relatively stable moral consciousness and behavior in the sports environment; this is sports moral character. From this perspective, sports moral character is composed of general moral character in individual life and special moral character in sports. General moral character is mainly affected by the family, school, and social environments. Special moral character in sports is derived from the individual in the process of sports activities, including sports competitions, games, fitness, etc. This characteristic of sports moral character can be called integration, which originates from the coordinated function of moral character and helps sports participants adjust their physical and mental state as they strive for moral goals incrementally through continual learning and development.

## 5. Analysis of the relationship between sports moral character and related concepts

In the previous research on and the elaboration of moral problems in sports, words such as sports moral character, sports morality, sportsmanship, etc. have commonly been used interchangeably. Although this variation in vocabulary is related to “users’” habits to a certain extent, it is also closely related to the essential properties of vocabulary and usage scenarios. Distinguishing among these words and usage scenarios can help to avoid confusion and to understand and solve the practical problems existing in vocabulary expressions.

### 5.1. Relationship between sports moral character and sports morality

Moral character is a stable psychological feature of moral values and norms that is gradually formed by individuals. Morality is a normative system that people form in their social life about the concepts, emotions and behavioral habits of good and evil, justice and partiality, and honesty and hypocrisy. Morality relies on the guidance of social public opinion and conscience to improve and regulate the relationships among humans and between humans and nature. In the field of sports, sports morality is the external environment in which individual sports moral character is formed. General societal norms, values, and practices in sport shape the environment, as does the media through television, movies, and newsprint (36, 37). Individual sports moral character helps improve the sports moral environment. The concepts are interrelated and interactive. For example, as an individual participates in sports, the moral qualities of fairness and integrity are gradually formed within the sports environment, with fair competition acting as the moral criterion. Here, fair competition is part of sports morality, while fairness and integrity are part of sports moral character. In addition, sports morality is seen as an objective “reason,” while sports moral character is seen as a subjective psychological characteristic. Therefore, when we discuss the cultivation of sports moral character, we should focus not only on the analysis and discussion of individual subjective values but also on the analysis of the sports ethics system. Spruit et al. (38) found that that in sports teams, the moral norms and values of coaches and team members, as well as the broader club culture, play extremely important roles in the moral behavior of young athletes. Escartí et al. (39) believed that the role of teachers in schools is very important. They must integrate physical activity and personal-social responsibility into each lesson, and gradually introduce the concept of transferring these responsibilities and life skills (Sport-based) to other settings.

In summary, sports morality and sports moral character are two different concepts. Sports morality can be regarded as the moral norm system present in the sports environment, while sports moral character is expressed as the personal moral quality present in the sports environment.

### 5.2. Relationship between sports moral character and sportsmanship

Sportsmanship is often used to describe moral character in sports. However, discussions of sportsmanship were initially used to describe the skills and abilities of those engaged in hunting activities and was only gradually adopted in competitive sports in the middle of the 19th century. At the same time, under the critical attitude of the gentleman class toward the competitive sports activities engaged in by other groups, the semantics and content of sportsmanship have become a vocabulary expressing ethics and norms in competitive sports (40). Subsequently, sportsmanship has become a synonym for morality in sports, and many researchers have defined sportsmanship in terms of moral character. Keating (41) noted that in sports activities and games, sportsmanship is a generous virtue, while in sports competition environments, it is a kind of fairness and justice. Abad (42) suggested that sportsmanship is about fairness; justice; good form; willingness to win; and, most importantly, maintaining a balance among all of these concepts. As a kind of virtue, sportsmanship has been widely recognized, but some scholars define sportsmanship from the perspective of character. Clifford and Feezell (43) believed that sportsmanship reflected not only a habit of respect but also an outstanding character. Shields and Bredemeier (44) regarded sportsmanship as a subconcept of sports character, which they defined as personal virtues such as empathy, fairness, sportsmanship, and integrity. In this regard, Shields believed that the research on sportsmanship was akin to that on moral value, which focuses on the content of moral thought.

However, sportsmanship cannot be completely equal to sports moral character. Lad Sessions (45) believed that sportsmanship and sports moral character are not the same thing. The moral code in sports does not constitute the whole of sportsmanship, while sports moral character is essentially moral. For example, we can use sportsmanship to evaluate the toughness of an athlete's will, but we cannot conclude that the athlete has good sports moral character. Moreover, in a study investigating teachers’ and students’ views on the concept of sportsmanship, Koç and Esentürk (46) found that teachers and students also regard “gentlemanship” and “making right decisions under tough conditions” as important contents of sportsmanship. In general, sportsmanship can be applied at the psychological and moral levels as well as to the expression of culture, thought, and ability in sports. Sportsmanship cannot be equated with sports moral character. Therefore, in the education and cultivation of sports moral character, we can use the rich content resources covered by sportsmanship, but we must explain the moral and non-moral factors contained in sportsmanship.

### 5.3. Relationship between sports moral character and sports character

Sports moral character and sports character are the presentation of “moral character” and “character” in sports. The difference in meaning between moral character and character has been discussed above. Moral character can be regarded as a subconcept of character, and in the sports context, sports moral character is a part of sports character. Sage (47) noted that sports character includes sports social character and sports moral character. These two branches also represent the differing views of two groups. The first group consists of coaches, managers, and players. They advocate teamwork, loyalty, self-sacrifice, and perseverance, which can be called the good characteristics and social values of “social character.” The second group is composed of sports scholars and the older generation. They usually look at character from a moral perspective and emphasize honesty, fairness, responsibility, compassion, and respect. Rudd and Stoll (7) used the Rudd–Stoll–Beller–Hahm value judgment scale to measure the social and moral character of athletes and nonathletes. The results showed that “professional sports training can cultivate social character, but not moral character.” From this point of view, it is certainly inappropriate to conclude that sports is not conducive to the development of sports character if the difference between sports character and sports moral character is ignored. For example, research reports have indicated a negative relationship between participation in sports and character development (44, 48, 49), but they have all discussed sports character at the moral level.

However, many studies have found a significant relationship between goal orientation and moral character (4, 50–52), and in the physical education environment, sports participants present a relatively more positive moral character (2, 53, 54). Weiss et al. (55) claimed that the potential of sport to improve youth participants’ perceptions of competence, relatedness, enjoyment, and self-determined motivation was highly dependent on the quality of interactions and relationships with important adults and peers. In this sense, when cultivating the sports moral character of sports participants, in addition to clarifying the differences between sports moral character, sports social character, and sports character, it is also necessary to make appropriate plans for the content and process of sports learning. The teaching personal and social responsibility model in physical education is a good example, which was considered to be one of the best models for promoting values, character, responsibility, and life skills in physical education and other physical activity settings (56, 57).

These differences between sports moral character and related concepts are shown in Table 1.

**Table 1:** Differences between sports moral character and related concepts.

Concept	Sports moral character and sports morality	Sports moral character and sportsmanship	Sports moral character and sports character
Characteristics	Sports moral character is expressed as the personal moral quality present in the sports environment, while sports morality can be regarded as the moral norm system present in the sports environment.	Sports moral character is essentially moral, while sportsmanship can be applied at the psychological and moral levels as well as to the expression of culture, thought, and ability in sports.	Sports moral character is part of sports character. Sports character includes not only moral character but also social character.

## 6. Conclusion

Based on the definition of moral character and the characteristics of sports moral character, this study proposes that the latter phrase refers to the stable moral quality that is gradually formed and displayed in sports practice through the influence of family, school, and social environments.

The concept of sports moral character is different from that of sports morality. Sports morality is the “reason” for an objective existence, while sports moral character is the psychological feature of individual “reason.” Moreover, sports character and sportsmanship are both more widely applied than is sports moral character, as these concepts are not limited to the moral level.

Furthermore, while this study focuses on the essence of sports moral character and related concepts, discussion remains lacking on the rational composition of sports morality and the education and evaluation methods of moral character. Thus, more research should be conducted in the future to discuss the standard system of sports morality and to further explore sports moral character education and evaluation methods.

## References

[1] Weiss MR Smith AL Stuntz CP. Moral development in sport and physical activity. In: Horn TS, editors. Advances in sport psychology. Champaign: Human Kinetics (2008). p. 187–210; 449–52.

[2] Shields DL Bredemeier BL. Advances in sport morality research. In: Tenenbaum G Eklund RC, editors. Handbook of sport psychology. Indianapolis: John Wiley & Sons, Inc (2007). p. 662–84.

[3] Narvaez D Lapsley DK. Moral identity, moral functioning, and the development of moral character. Psychol Learn Motiv. (2009) 50:237–74. 10.1016/S0079-7421(08)00408-8

[4] Jang CY. Development and validation of the sport character scale. Utah: The University of Utah (2013).

[5] Konch M Panda RK. Aristotle on habit and moral character formation. Int J Ethics Educ. (2019) 4(1):31–41. 10.1007/s40889-018-0061-7

[6] Morse L Cohen TR. Moral character in negotiation. Acad Manag Perspect. (2019) 33(1):12–25. 10.5465/amp.2017.0051

[7] Rudd A Stoll SK. What type of character do athletes possess? An empirical examination of college athletes versus college non athletes with the RSBH value judgment inventory. Sport J. (2004) 7(2).

[8] Ameriks K Clarke DM. Aristotle: Nicomachean ethics. England: Cambridge University Press (2000).

[9] George MI. What moral character is and is not. Linacre Q. (2017) 84(3):261–74. 10.1080/00243639.2017.1338442 28912619 PMC5592308

[10] Pervin LA. A critical analysis of current trait theory. Psychol Inq. (1994) 5(2):103–13. 10.1207/s15327965pli0502_1

[11] Cohen TR Morse L. Moral character: what it is and what it does. Res Organ Behav. (2014) 34:43–61. 10.1016/j.riob.2014.08.003

[12] Gert B Gert J. The definition of morality. In: Zalta EN, editors. The Stanford encyclopedia of philosophy. Stanford: Metaphysics Research Lab, Stanford University (2020).

[13] Wu JL. On “morality”—moral concept and definition ideas (in Chinese). J Jiangxi Norm Univ. (2011) 44(01):36–42. 10.3969/j.issn.1000-579X.2011.01.004

[14] Simon B. A dictionary of philosophy. 3rd ed. Oxford: Oxford University Press (2016).

[15] Lickona T. The return of character education. Educ Leadersh. (1993) 51(3):6–11.

[16] Berkowitz MW Grych JH. Early character development and education. Early Educ Dev. (2000) 11(1):55–72. 10.1207/s15566935eed1101_4

[17] Lickona T Davidson M. Smart & good high schools: integrating excellence and ethics for success in school, work, and beyond. Center for the 4th and 5th Rs/Character Education Partnership (2005).

[18] Shields DL. Character as the aim of education. Phi Delta Kappan. (2011) 92(8):48–53. 10.1177/003172171109200810

[19] Baehr J. The varieties of character and some implications for character education. J Youth Adolesc. (2017) 46(6):1153–61. 10.1007/s10964-017-0654-z 28332052

[20] Lin PJ. A translation of Lao-tzu’s tao te ching and wang pi’s commentary. Michigan: University of Michigan Press (2020).

[21] Legge J. The I Ching (vol. 16). New York: Courier Corporation (1963).

[22] Logic teaching and research office of philosophy department of East China Normal University. Formal logic (in Chinese), East China Normal University Press (2016).

[23] Ma JK Le ML Gurd BJ. Four minutes of in-class high-intensity interval activity improves selective attention in 9-to-11-year-olds. Appl Physiol Nutr Metab. (2015) 40(3):238–44. 10.1139/apnm-2014-0309 25675352

[24] De Greeff JW Bosker RJ Oosterlaan J Visscher C Hartman E. Effects of physical activity on executive functions, attention and academic performance in preadolescent children: a meta-analysis. J Sci Med Sport. (2018) 21(5):501–7. 10.1016/j.jsams.2017.09.595 29054748

[25] Jiménez-Parra JF Belando-Pedreño N Valero-Valenzuela A. The effects of the active values program on psychosocial aspects and executive functions. Int J Environ Res Public Health. (2023) 20(1):595. 10.3390/ijerph20010595 PMC9819049 36612915

[26] Hellison D Martinek T. Social and personal responsibility programs. In: Kirk D O’Sullivan M MacDonald D, editors. Handbook of research in physical education. London, UK: Sage (2006). p. 610–26.

[27] Courel-Ibáñez J Sánchez-Alcaraz BJ Gomez-Marmol A Valero-Valenzuela A Moreno-Murcia JA. The moderating role of sportsmanship and violent attitudes on social and personal responsibility in adolescents. A clustering-classification approach. PloS One. (2019) 14(2):e0211933. 10.1371/journal.pone.0211933 30730958 PMC6366707

[28] Brenda LB Shields DL. Sports and character development. J Phys Act Health. (2006) 7:1–8. 10.1123/jpah.3.2.255 28834461

[29] Yaffe Y Levental O Arey DL Lev A. Morality and values in sports among young athletes: the role of sport type and parenting styles: a pilot study. Front Psychol. (2021) 12:618507. 10.3389/fpsyg.2021.618507 33643147 PMC7902507

[30] Sanlav R Donuk B. Examination of the effects of individual and team athletes regarding sports character sub-dimensions differences. J Soc Humanit Adm Sci. (2021) 7:35. 10.31589/JOSHAS.507

[31] Suwiwa IG Sutajaya IM Sudiarta IGP Wahjoedi W Swadesi IKI. 21st Century character education for Indonesian children through sport science. Jurnal Kependidikan. (2022) 8(1):68–76. 10.33394/jk.v8i1.4265

[32] Burgueño R Medina-Casaubón J. Sport education and sportsmanship orientations: an intervention in high school students. Int J Environ Res Public Health. (2020) 17(3):837. 10.3390/ijerph17030837 32013106 PMC7036946

[33] Laker A. Developing personal, social and moral education through physical education: A practical guide for teachers. London: Routledge (2002).

[34] Piaget J. The moral judgment of the child. Simon & Schuster (1997).

[35] Kohlberg L. Stages of moral development. Moral Educ. (1971) 1(51):23–92. 10.3138/9781442656758-004

[36] Stoll SK Beller JM. Do sports build character? In: Gerdy JR, editors. Sports in school: 372 the future of an institution. New York: Teachers College Press (2000). p. 18–30.

[37] Pennington CG. Moral development and sportsmanship in physical education and sport. J Phys Educ Recreat Dance. (2017) 88(9):36–42. 10.1080/07303084.2017.1367745

[38] Spruit A Kavussanu M Smit T Ijntema M. The relationship between moral climate of sports and the moral behavior of young athletes: a multilevel meta-analysis. J Youth Adolesc. (2019) 48(2):228–42. 10.1007/s10964-018-0968-5 30560510 PMC6394495

[39] Escartí A Gutiérrez M Pascual C Llopis R. Implementation of the personal and social responsibility model to improve self-efficacy during physical education classes for primary school children. Int J Psychol Psychol Ther. (2010) 10(3):387–402.

[40] Lei P. Ethics, politics and sportsmanship: the meaning of British sportsmanship in the nineteenth century (in Chinese). Chi Sports Sci Technol. (2015) 51(3):121–6; +130. 10.16470/j.csst.201503017

[41] Keating JW. Sportsmanship as a moral category. Ethics. (1964) 75(1):25–35. 10.1086/291517

[42] Abad D. Sportsmanship. Sport Ethics Philos. (2010) 4(1):27–41. 10.1080/17511320903365227

[43] Clifford C Feezell RM. Sport and character: Reclaiming the principles of sportsmanship. Champaign: Ringgold, Inc (2010); Reference & Research Book News, 25(2).

[44] Shields DL Bredemeier BL. Character development and physical activity. Champaign: Human Kinetics Publishers (1995).

[45] Lad Sessions W. Sportsmanship as honor. J Philos Sport. (2004) 31(1):47–59. 10.1080/00948705.2004.9714648

[46] Koç Y Esentürk OK. Opinions of physical education teachers on the concept of sportsmanship. J Educ Learn. (2018) 7(1):71–9. 10.5539/jel.v7n1p71

[47] Sage G. Does sport affect character development in athletes? J Phys Educ Recreat Dance. (1998) 69(1):15–8. 10.1080/07303084.1998.10605041

[48] Silva J. The perceived legitimacy of rule violating behavior in sport. J Sport Psychol. (1983) 5:438–48. 10.1123/jsp.5.4.438

[49] Krause J Priest R. Sport value choices of U.S. military cadets—a longitudinal study of the class of 1993. Unpublished manuscript, Office of Institutional Research, U.S. Military Academy. West Point, NY (1993).

[50] Lemyre PN Roberts GC Ommundsen Y. Achievement goal orientations, perceived ability, and sportspersonship in youth soccer. J Appl Sport Psychol. (2002) 14:120–36. 10.1080/10413200252907789

[51] Kavussanu M. Motivational predictors of prosocial and antisocial behavior in football. J Sports Sci. (2006) 24(6):575–88. 10.1080/02640410500190825 16611569

[52] Sage L Kavussanu M. The effects of goal involvement on moral behavior in an experimentally manipulated competitive setting. J Sport Exerc Psychol. (2007) 29:190–207. 10.1123/jsep.29.2.190 17568066

[53] Romance TJ Weiss MR Bockoven J. A program to promote moral development through elementary school physical education. J Teach Phys Educ. (1986) 5:126–36. 10.1123/jtpe.5.2.126

[54] Strachan L McHugh TL Mason C. Understanding positive youth development in sport through the voices of indigenous youth. J Sport Exerc Psychol. (2018) 40(6):293–302. 10.1123/jsep.2018-0035 30517819

[55] Weiss MR Kipp LE Espinoza SM. Motivational processes in youth sport and physical activity. In: Ryan RM, editors. The Oxford handbook of human motivation. Oxford: Oxford University Press (2019). p. 487–506.

[56] Hellison D. Teaching responsibility through physical activity. 3rd ed Champaign, IL: Human Kinetics (2011).

[57] Pozo P Grao-Cruces A Pérez-Ordás R. Teaching personal and social responsibility model-based programmes in physical education: a systematic review. Eur Phy Educ Rev. (2018) 24(1):56–75. 10.1177/1356336X16664749

